# ﻿The taxonomy of *Cyrtodactylusconsobrinus* (Peters, 1871) (Squamata, Gekkonidae) and the description of a new species from the Thai-Malay Peninsula

**DOI:** 10.3897/zookeys.1241.149552

**Published:** 2025-06-12

**Authors:** L. Lee Grismer, Amanda Kaatz, Jesse L. Grismer, Eddie Nguyen, Jeren J. Grergory, Perry L. Wood Jr., Matthew L. Murdoch, Shahrul Anuar, Chan Kin Onn, Muhamad A. Muin, Parinya Pawangkhanant, Chatmongkon Suwannapoom, Nikolay A. Poyarkov, Evan S. H. Quah

**Affiliations:** 1 Herpetology Laboratory, Department of Biology, La Sierra University, 4500 Riverwalk Parkway, Riverside, California 92505, USA La Sierra University Riverside United States of America; 2 Department of Herpetology, San Diego Natural History Museum, PO Box 121390, San Diego, California, 92112, USA Universiti Malaysia Sabah Kota Kinabalu Malaysia; 3 Institute for Tropical Biology and Conservation, Universiti Malaysia Sabah, Jalan UMS, 88400, Kota Kinabalu, Malaysia Department of Herpetology, San Diego Natural History Museum San Diego United States of America; 4 Department of Ecology and Evolutionary Biology, University of Michigan, Ann Arbor, MI 48103, USA University of Michigan Ann Arbor United States of America; 5 School of Biological Sciences, Universiti Sains Malaysia, 11800 Minden, Penang, Malaysia Universiti Sains Malaysia Minden Malaysia; 6 University of Kansas Biodiversity Institute and Natural History Museum, Lawrence, KS 66045, USA University of Kansas Biodiversity Institute and Natural History Museum Lawrence United States of America; 7 Division of Fishery, School of Agriculture and Natural Resources, University of Phayao, Phayao 56000, Thailand University of Phayao Phayao Thailand; 8 Rabbit in the Moon Foundation, Suanphueng, Ratchaburi 70180, Thailand Rabbit in the Moon Foundation Ratchaburi Thailand; 9 Department of Vertebrate Zoology, Lomonosov Moscow State University, Leninskiye Gory, Moscow 119234, Russia Lomonosov Moscow State University Moscow Russia; 10 Joint Vietnam - Russia Tropical Science and Technology Research Center, 63 Nguyen Van Huyen Road, Nghia Do, Cau Giay, Hanoi 122000, Vietnam Joint Vietnam - Russia Tropical Science and Technology Research Center Hanoi Vietnam; 11 Lee Kong Chian Natural History Museum, National University of Singapore, 2 Conservatory Drive, 117377, Singapore, Singapore National University of Singapore Singapore Singapore

**Keywords:** Bent-toed Gecko, Borneo, integrative taxonomy, Peninsular Malaysia, phylogeny, Thailand

## Abstract

Phylogenetic analyses based on 1459 base pairs of the mitochondrial gene ND2 and its flanking tRNAs indicate that *Cyrtodactylusconsobrinus* from the type locality in Sarawak, East Malaysia (Borneo) and *C.consobrinus* from Peninsular Malaysia are not conspecific. Both populations as well as *C.hutan* from East Malaysia form a strongly supported monophyletic group even though their relationships to one another remain unresolved. *Cyrtodactylusconsobrinus* from peninsular Malaysia is described herein as the new species *C.peninsularis***sp. nov.** whose type locality is Gunung Belumut, Johor State. *Cyrtodactyluspeninsularis***sp. nov.** is diagnosable from all other species in the *malayanus* group by having statistically different morphospatial positions in multiple factor analyses (MFA) based on size-corrected morphometric and meristic characters. ANOVA analyses of these characters recovered significantly different mean values between *C.peninsularis***sp. nov.** and varying combinations of all other *malayanus* group species across several size-corrected morphometric and meristic characters. Genetic variation within *C.peninsularis***sp. nov.** is geographically structured across six well supported monophyletic mitochondrial lineages bearing an uncorrected pairwise sequence divergence ranging from 0.97–4.5%. Despite its well supported phylogeographic structure, PCAs and ANOVAs recovered statistically weak morphological separation among the lineages and as such, all are considered conspecific pending a genomic analysis. The phylogeographic structure within the forest-dwelling *C.peninsularis***sp. nov.** is quite similar to that of the stream-adapted ranid frog genus *Amolops* and less so to that of the microhabitat specialists of the *C.pulchellus* group and the forest generalist *C.quadrivirgatus*, all of whom are sympatric across Peninsular Malaysia.

## ﻿Introduction

The *Cyrtodactylusmalayanus* group contains some of the most remarkedly patterned Bent-toed geckos that range throughout the primary and secondary forests of Sundaland (sec. Grismer et al. 2021). The group embodies a broad array of adaptive types as well, from tree trunk and shrub dwellers to karst associated specialists (Grismer 2011; Davis et al. 2023). At least three of the five species of the *malayanus* group are endemic to Borneo (Davis et al. 2019, 2021) and one, *C.consobrinus* (Peters, 1871), ranges widely outside Borneo having been reported from Thailand, Peninsular Malaysia, Singapore (Grismer 2011), and the Indonesian Island of Singkep (de Rooij 1915). In a recent phylogenetic analysis of the *malayanus* group (Davis et al. 2023), populations of *C.consobrinus* from the Thai-Malay Peninsula and Borneo were recovered as sister lineages but with weak nodal support. The analysis was based on 1018 of the mtDNA gene fragment ND2 but included only two samples of *C.consobrinus* from southwestern Peninsular Malaysia. To test the hypothesis that these populations are not conspecific, we increased the sample size of peninsular *C.consobrinus* to 27, covering its range from southern Thailand through Peninsular Malaysia. We also used a 1459 base pair gene fragment of ND2 and its flanking tRNAs. Univariate and multivariate analyses were employed on the limited morphological data sets in Davis et al. (2020, 2023) to search for statistically significant diagnostic differences between peninsular *C.consobrinus* and Bornean *C.consobrinus* as well as among the other species of the *malayanus* group.

**Figure 1.**
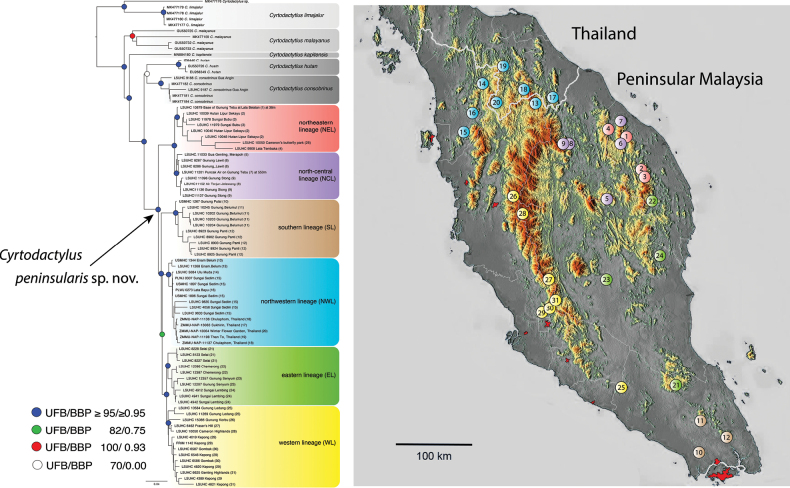
Maximum likelihood consensus topology based on 1459 base pairs of ND2 and flanking tRNAs showing the relationships among the species of the *malayanus* group and among the lineages within *Cyrtodactyluspeninsularis* sp. nov. Numbered individuals of *C.peninsularis* sp. nov. in the tree match the locality numbers on the distribution map of the six lineages on the Thai-Malay peninsula.

Phylogenetic analyses were employed to test for population-level structure within peninsular *C.consobrinus* across its entire range (Fig. 1). In corroboration, an expanded morphological data set composed of 31 characters and 52 specimens from throughout its range on the Thai-Malay Peninsula was analyzed in order to compare phylogeographic structure with geographic variation in morphology. Integrating across the results of all these analyses indicate that peninsular and Bornean *C.consobrinus* are not conspecific and the notable phylogenetic substructure within peninsular *C.consobrinus* is not geographically concordant with its weak geographic morphological variation. Based on this, and the fact that the type locality for *C.consobrinus* is “Sarawak [Borneo]” (Peters 1871: 569), we describe peninsular *C.consobrinus* as the new species *C.peninsularis*.

## ﻿Materials and methods

### ﻿Molecular data

Liver samples taken from 69 specimens of *Cyrtodactylusconsobrinus* throughout its range in southern Thailand and Peninsular Malaysia were stored in 95% ethanol. Genomic DNA was isolated from liver or skeletal muscle specimens stored in 95% ethanol using the Qiagen DNeasy animal tissue kit (Valencia, CA, USA). The mitochondrial NADH dehydrogenase subunit 2 gene (**ND2**) was amplified using a double-stranded polymerase chain reaction (PCR) under the following conditions: 1.0 μL genomic DNA (10–30 μg), 1.0 μL light strand primer (concentration 10 μM), 1.0 μL heavy strand primer (concentration 10 μM), 1.0 μL dinucleotide pairs (1.5 μM), 2.0 μL 5 × buffer (1.5 μM), MgCl 10 × buffer (1.5 μM), 0.1 μL Taq polymerase (5 U/μL) and 6.4 μL ultra-pure H2O. PCR reactions were executed on an Eppendorf Mastercycler gradient thermocycler under the following conditions: initial denaturation at 95 °C for 2 min, followed by a second denaturation at 95 °C for 35 s, annealing at 48–50 °C for 35 s, followed by a cycle extension at 72 °C for 35 s, for 31 cycles. All PCR products were visualized on a 1.0% agarose gel electrophoresis. Successful PCR products were vacuum purified using MANU 30 PCR plates (Millipore) and purified products were resuspended in ultra-pure water. Purified PCR products were sequenced using the ABI Big-Dye Terminator v3.1 Cycle Sequencing Kit in an ABI GeneAmp PCR 9700 thermal cycler. Cycle sequencing reactions were purified with Sephadex G-50 Fine (GE Healthcare) and sequenced on an ABI 3730xl DNA Analyzer at the BYU (Brigham Young University) DNA Sequencing Centre (DNASC). Primers used for amplification are L4437b Macey et al. (1997) External 5′-AAGCAGTTGGGCCCATACC-3′ and H5934 (Macey et al. 1997) External 5′-AGRGTGCCAATGTCTTTGTGRTT-3′ and we also used these for sequencing along with two internal primers CyrtintF1 Siler et al. (2010) Internal 5′-TAGCCYTCTCYTCYATYGCCC-3′ CyrtintR1 Siler et al. (2010) Internal 5′-ATTGTKAGDGTRGCYAGGSTKGG-3′. To this, four samples of *C.limajalur* Davis, Bauer, Jackman, Nashriq & Das, 2019, four samples of *C.malayanus* (de Rooij, 1915), one sample of *C.kapitensis* Davis, Nashriq, Woytek, Wikramanayake, Bauer, Karin, Brennan, Iskandar & Das, 2023, three samples of *C.hantu* Davis, Das, Leaché, Karin, Brennan, Jackman, Nashriq, Chan & Bauer, 2021, and one sample of an unnamed species of the *malayanus* group were downloaded from GenBank (Table 1). The protein-coding region of the ND2 sequence was aligned manually. Mesquite (Maddison and Maddison 2015) was used to calculate the correct amino acid reading frame and to confirm the lack of premature stop codons. The GenBank accession numbers for all specimens are listed in Table 1.

**Table 1.** Voucher specimens and GenBank accession numbers for ND2 and its flanking tRNAs.

Species	Voucher specimen	Locality	GenBank number
*Cyrtodactylushutan*	Davis et al. (2021)	East Malaysia, Sarawak, Kapit Division, Nanga Merit	ID8440
*Cyrtodactylushutan*	Davis et al. (2021)	East Malaysia, Sarawak, Kapit Division, Nanga Merit	GU550726
*Cyrtodactylushutan*	Davis et al. (2021)	East Malaysia, Sarawak, Kapit Division, Nanga Merit	EU268349
*Cyrtodactyluskapitensis*	Davis et al. (2023)	East Malaysia, Sarawak, Kapit Division, Pelagus NP	MN884180
*Cyrtodactyluslimajalur*	Davis et al. (2019)	East Malaysia, Sarawak, Serian, Kampung Tubih Mawang	MK477179
*Cyrtodactyluslimajalur*	Davis et al. (2019)	East Malaysia, Sarawak, Serian, Kampung Tubih Mawang	MK477178
*Cyrtodactyluslimajalur*	Davis et al. (2019)	East Malaysia, Sarawak, Serian, Kampung Tubih Mawang	MK477160
*Cyrtodactyluslimajalur*	Davis et al. (2019)	East Malaysia, Sarawak, Serian, Kampung Tubih Mawang	MK477177
*Cyrtodactylusmalayanus*	Siler et al. (2010)	East Malaysia, Sarawak	GU550725
*Cyrtodactylusmalayanus*	Siler et al. (2010)	East Malaysia, Sarawak	MK477159
*Cyrtodactylusmalayanus*	Siler et al. (2010)	East Malaysia, Sarawak	GU550732
*Cyrtodactylusmalayanus*	Siler et al. (2010)	East Malaysia, Sarawak	GU550733
*Cyrtodactylusconsobrinus*	Davis et al. (2019)	East Malaysia, Sarawak	MK477182
*Cyrtodactylusconsobrinus*	Davis et al. (2019)	East Malaysia, Sarawak	MK477181
*Cyrtodactylusconsobrinus*	Davis et al. (2019)	East Malaysia, Sarawak	MK477184
*Cyrtodactylusconsobrinus*	LSUHC 9188	East Malaysia, Sarawak, Gua Angin	PQ629996
*Cyrtodactylusconsobrinus*	LSUHC 9187	East Malaysia, Sarawak, Gua Angin	PQ629995
*Cyrtodactyluspeninsularis* sp. nov.	PLWJ 307	West Malaysia, Kedah, Bailing, Lata Bayu	PQ629968
*Cyrtodactyluspeninsularis* sp. nov.	PLWJ 273	West Malaysia, Kedah, Sungai Sedim	PQ629967
*Cyrtodactyluspeninsularis* sp. nov.	LSUHC 9908	West Malaysia, Terengganu, Lata Tembaka	PQ629961
*Cyrtodactyluspeninsularis* sp. nov.	LSUHC 9835	West Malaysia, Kedah, Sungai Sedim	PQ629960
*Cyrtodactyluspeninsularis* sp. nov.	LSUHC 9633 paratype	West Malaysia, Kedah, Sungai Sedim	PQ629959
*Cyrtodactyluspeninsularis* sp. nov.	LSUHC 8925	West Malaysia, Johor, Gunung Panti FR, Bunker Trail	PQ629958
*Cyrtodactyluspeninsularis* sp. nov.	LSUHC 8924	West Malaysia, Johor, Gunung Panti FR, Bunker Trail	PQ629957
*Cyrtodactyluspeninsularis* sp. nov.	LSUHC 8923	West Malaysia, Johor, Gunung Panti FR, Bunker Trail	PQ629956
*Cyrtodactyluspeninsularis* sp. nov.	LSUHC 8903	West Malaysia, Johor, Gunung Panti FR, Bunker Trail	PQ629955
*Cyrtodactyluspeninsularis* sp. nov.	LSUHC 8902	West Malaysia, Johor, Gunung Panti FR, Bunker Trail	PQ629954
*Cyrtodactyluspeninsularis* sp. nov.	LSUHC 8287	West Malaysia, Terengganu, Gunung Tebu	PQ629953
*Cyrtodactyluspeninsularis* sp. nov.	LSUHC 8286	West Malaysia, Terengganu, Gunung Tebu	PQ629952
*Cyrtodactyluspeninsularis* sp. nov.	LSUHC 8228	West Malaysia, Johor, Selai, Lubuk Tapah	PQ629951
*Cyrtodactyluspeninsularis* sp. nov.	LSUHC 8227	West Malaysia, Johor, Selai, Lubuk Tapah	PQ629950
*Cyrtodactyluspeninsularis* sp. nov.	LSUHC 8123	West Malaysia, Johor, Selai, Lubuk Tapah	PQ629949
*Cyrtodactyluspeninsularis* sp. nov.	LSUHC 6625	West Malaysia, Pahang, Genting highlands	PQ629948
*Cyrtodactyluspeninsularis* sp. nov.	LSUHC 6587	West Malayisa, Selangor, Gombak	PQ629973
*Cyrtodactyluspeninsularis* sp. nov.	LSUHC 6546	West Malaysia, Selangor, Kepong, FRIM	PQ629947
*Cyrtodactyluspeninsularis* sp. nov.	LSUHC 6462	West Malaysia, Pahang, Fraser’s Hill, the Gap	PQ629946
*Cyrtodactyluspeninsularis* sp. nov.	LSUHC 5084	West Malaysia, Kedah, Ulu Muda, Gubir	PQ629990
*Cyrtodactyluspeninsularis* sp. nov.	LSUHC 4942	West Malaysia, Pahang, Sungai Lembing Logging Camp	PQ629982
*Cyrtodactyluspeninsularis* sp. nov.	LSUHC 4941	West Malaysia, Pahang, Sungai Lembing Logging Camp	PQ629945
*Cyrtodactyluspeninsularis* sp. nov.	LSUHC 4912	West Malaysia, Pahang, Sungai Lembing Logging Camp	PQ629944
*Cyrtodactyluspeninsularis* sp. nov.	LSUHC 4821	West Malaysia, Selangor, Kepong, FRIM	PQ629943
*Cyrtodactyluspeninsularis* sp. nov.	LSUHC 4820	West Malaysia, Selangor, Kepong, FRIM	PQ629942
*Cyrtodactyluspeninsularis* sp. nov.	LSUHC 4389	West Malaysia, Selangor, Kepong, FRIM	PQ629941
*Cyrtodactyluspeninsularis* sp. nov.	LSUHC 4056	West Malaysia, Kedah, Sungai Sedim	PQ629940
*Cyrtodactyluspeninsularis* sp. nov.	LSUHC 4019	West Malaysia, Selangor, Kepong, FRIM	PQ629939
*Cyrtodactyluspeninsularis* sp. nov.	LSUHC 15086	West Malaysia, Perak, Gunung Korbu	PQ629992
*Cyrtodactyluspeninsularis* sp. nov.	LSUHC 12387	West Malaysia, Terengganu, Hutan Lipur Chemerong	PQ629938
*Cyrtodactyluspeninsularis* sp. nov.	LSUHC 12386 paratype	West Malaysia, Terengganu, Hutan Lipur Chemerong	PQ629937
*Cyrtodactyluspeninsularis* sp. nov.	LSUHC 12357	West Malayisa, Pahang, Hutan Lipur Gunung Senyum	PQ629980
*Cyrtodactyluspeninsularis* sp. nov.	LSUHC 12207	West Malayisa, Pahang, Hutan Lipur Gunung Senyum	PQ629983
*Cyrtodactyluspeninsularis* sp. nov.	LSUHC 11979 paratype	West Malaysia, Terengganu, Sungai Bubu	PQ629989
*Cyrtodactyluspeninsularis* sp. nov.	LSUHC 11978	West Malaysia, Terengganu, Sungai Bubu	PQ629988
*Cyrtodactyluspeninsularis* sp. nov.	LSUHC 11269	West Malaysia, Johor, Gunung Ledang	PQ629994
*Cyrtodactyluspeninsularis* sp. nov.	LSUHC 11267 paratype	West Malaysia, Perak, Belum, Sungai Enam	PQ629991
*Cyrtodactyluspeninsularis* sp. nov.	LSUHC 11201	West Malaysia, Terengganu, Gunung Tebu, Punca Air	PQ629966
*Cyrtodactyluspeninsularis* sp. nov.	LSUHC 11152 paratype	West Malaysia, Kelantan, Hutan Lipur Jelawang	PQ629965
*Cyrtodactyluspeninsularis* sp. nov.	LSUHC 11137 paratype	West Malaysia, Kelantan, Gunung Stong	PQ629964
*Cyrtodactyluspeninsularis* sp. nov.	LSUHC 11136 paratype	West Malaysia, Kelantan, Gunung Stong	PQ629963
*Cyrtodactyluspeninsularis* sp. nov.	LSUHC 11096	West Malaysia, Kelantan,Gunung Stong, Kem Baha	PQ629962
*Cyrtodactyluspeninsularis* sp. nov.	LSUHC 11033	West Malaysia, Pahang, Merapoh, Gua Gunting	PQ629981
*Cyrtodactyluspeninsularis* sp. nov.	LSUHC 10879	West Malaysia, Terengganu, base of Gunung Tebu	PQ629936
*Cyrtodactyluspeninsularis* sp. nov.	LSUHC 10584	West Malaysia, Johor, Gunung Ledang	PQ629935
*Cyrtodactyluspeninsularis* sp. nov.	LSUHC 10245	West Malaysia, Johor, Gunung Belumut, 245 m	PQ629984
*Cyrtodactyluspeninsularis* sp. nov.	LSUHC 10204	West Malaysia, Johor, base of Gunung Belumut	PQ629986
*Cyrtodactyluspeninsularis* sp. nov.	LSUHC 10230 paratype	West Malaysia, Johor, base of Gunung Belumut	PQ629979
*Cyrtodactyluspeninsularis* sp. nov.	LSUHC 10202 holotype	West Malaysia, Johor, base of Gunung Belumut	PQ629985
*Cyrtodactyluspeninsularis* sp. nov.	LSUHC 10050	West Malaysia, Pahang, Cameron Highlands	PQ629934
*Cyrtodactyluspeninsularis* sp. nov.	LSUHC 10050	West Malaysia, Pahang, Cameron Highlands	PQ629993
*Cyrtodactyluspeninsularis* sp. nov.	LSUHC 10048 paratype	West Malaysia, Terengganu, Hutan Lipur Sekayu	PQ629933
*Cyrtodactyluspeninsularis* sp. nov.	LSUHC 10040	West Malaysia, Terengganu, Hutan Lipur Sekayu	PQ629932
*Cyrtodactyluspeninsularis* sp. nov.	LSUHC 10039	West Malaysia, Terengganu, Hutan Lipur Sekayu	PQ629987
*Cyrtodactyluspeninsularis* sp. nov.	USMHC 1897	West Malaysia, Kedah, Sungai Sedim	PQ629972
*Cyrtodactyluspeninsularis* sp. nov.	USMHC 1896	West Malaysia, Kedah, Sungai Sedim	PQ629971
*Cyrtodactyluspeninsularis* sp. nov.	USMHC 1344	West Malaysia, Perak, Belum, Sungai Enam	PQ629970
*Cyrtodactyluspeninsularis* sp. nov.	USMHC 1267	West Malaysia, Johor, Gunung Pulai	PQ629969
*Cyrtodactyluspeninsularis* sp. nov.	ZMMU-NAP 13064	Thailand, Yala, Winter Flower Garden	PQ629978
*Cyrtodactyluspeninsularis* sp. nov.	ZMMU-NAP 13063	Thailand, Narathiwat, Sukhirin	PQ629974
*Cyrtodactyluspeninsularis* sp. nov.	ZMMU-NAP 11198	Thailand, Narathiwat, Chulaphom	PQ629977
*Cyrtodactyluspeninsularis* sp. nov.	ZMMU-NAP 11137	Thailand, Yala, Than To	PQ629976

### ﻿Phylogenetic analyses

Maximum likelihood (**ML**) and Bayesian inference (**BI**) were used to estimate the phylogenetic relationships based on aligned sequences. The ML phylogeny was estimated using the IQ-TREE webserver (Nguyen et al. 2015; Trifinopoulos et al. 2016) preceded by the selection of the best substitution models using the Bayesian Information Criterion (**BIC**) in ModelFinder (Kalyaanamoorthy et al. 2017), which supported K2P+I+G4 for the non-coding position, TIM3+F+I+G4 for codon position1, TN+F+I+G4 for codon position 2, and TVM+F+T+G4 for codon position 3. Ten-thousand bootstrap pseudoreplicates via the ultrafast bootstrap (UFB; Hoang et al. 2018) approximation algorithm were employed and nodes having UFB values of 95 and above were considered strongly supported (Minh et al. 2013). The Bayesian inference (BI) analysis was carried out in MrBayes 3.2.3. (Ronquist et al. 2012) on XSEDE using the CIPRES Science Gateway (Cyberinfrastructure for Phylogenetic Research; Miller et al. 2010) employing bModelTest for codon partitioning. Two independent Markov chain Monte Carlo (**MCMC**) simulations were performed each with four chains, three hot and one cold. We ran the MCMC simulation for 50 million generations, sampled every 5,000 generations, and discarded the first 10% of each run as burn-in. Convergence and stationarity of all parameters from both runs were checked in Tracer v1.6 (Rambaut and Drummond 2013) to ensure effective sample sizes (**ESS**) were above 200. Post-burn-in sampled trees from both runs were combined using the sumt function and a 50% majority rule consensus tree was constructed. Nodes with Bayesian posterior probabilities (**BPP**) of 0.95 and above were considered strongly supported (Huelsenbeck et al. 2001; Wilcox et al. 2002). Retaining only ingroup taxa, uncorrected pairwise sequence divergences were calculated in MEGA X (Tamura et al. 2021) using the pairwise deletion option.

### ﻿Morphological data

#### ﻿Morphometric characters

Measurements were taken on the left side of the body to the nearest 0.1 mm using Mitutoyo dial calipers under a Nikon SMZ 1500 dissecting microscope and follow Grismer and Grismer (2017) and Grismer et al. (2018). They included: snout-vent length (**SVL**), taken from the tip of the snout to the vent; tail length (**TL**), taken from the vent to the tip of the tail; tail width (**TW**), taken at the base of the tail immediately posterior to the postcloacal swelling; brachial length (**BracL**), taken from the insertion of the humerus in the glenoid fossa to the posterior margin of the elbow while flexed 90°; forearm length (**ForeL**), taken on the ventral surface from the posterior margin of the elbow while flexed 90° to the inflection of the flexed wrist; femoral (thigh) length (**FemL**), taken from the insertion of the femur in the acetabulum to the posterior margin of the knee while flexed 90°; tibia length (**TibL**), taken on the ventral surface from the posterior surface of the knee while flexed 90° to the base of the heel; axilla to groin length (**AG**), taken from the posterior margin of the forelimb at its insertion point on the body to the anterior margin of the hind limb at its insertion point on the body; head length (**HL**), the distance from the posterior margin of the retroarticular process of the lower jaw to the tip of the snout; head width (**HW**), measured at the angle of the jaws; head depth (**HD**), the maximum height of head measured from the occiput to base of the lower jaw; eye diameter (**ED**), the greatest horizontal diameter of the eye-ball; eye to ear distance (**EE**), measured from the anterior edge of the ear opening to the posterior edge of the bony orbit; snout length (**SN**), measured from anteriormost margin of the bony orbit to the tip of snout; eye to nostril distance (**EN**), measured from the anterior margin of the bony orbit to the posterior margin of the external naris; interorbital distance (**IO**), measured between the anterior-most edges of the bony orbits; ear length (**EarL**), measured as the greatest vertical distance of the ear opening; and internarial distance (**IN**), measured between the nares across the rostrum.

#### ﻿Meristic characters

Scale and precloacal pore counts taken were supralabial scales (**SL**) counted from the largest scale immediately below the eyeball to the rostral scale; infralabial scales (**IL**) counted from the mental to the termination of enlarged scales just after the upturn of the mouth; number of internasal scales (**INA**) counted between the external nares; the number of paravertebral tubercles (**PVT**) between limb insertions counted in a straight line immediately left or right of the vertebral column; the number of longitudinal rows of body tubercles (**LRT**) counted transversely across the center of the dorsum from one ventrolateral fold to the other; the number of longitudinal rows of ventral scales (**LVS**) counted transversely across the center of the abdomen from one ventrolateral fold to the other; transverse rows of ventral scales (**TVS**), counted from the postmentals ending with the large scales bordering the granular scales anterior to the vent; the number of expanded subdigital lamellae on the fourth toe (**E4TL**) counted from the base of the first phalanx to the large scale on the digital inflection; the number of unexpanded subdigital lamellae on the fourth toe (**U4TL**) counted from the digital inflection to the end of the digit and including the claw sheath; the total number of subdigital lamellae on the fourth toe (**T4TL = E4TL+U4TL**) counted from the base of the first phalanx where it contacts the body of the foot to the claw and including the claw sheath (see Murdoch et al. 2019: fig. 2); number of enlarged femoral scales (**FS**); total number of enlarged femoral scales from each thigh (**TFS**). In some species, only the distalmost FS on each side are greatly enlarged, and the proximal scales are smaller whereas in others, the enlarged scales are continuous with the enlarged precloacal scales. The separation of the two scales rows was determined to be at a point even with the lateral body margin (see Murdoch et al. 2019: fig. 3). The number of enlarged precloacal scales (**PS**); the total number of femoral pores in males (**FP**); the number of precloacal pores in (**PP**) in males and some females; the number of rows of enlarged post-precloacal scales (**PPS**) on the midline between the enlarged precloacal scales and the granular scales anterior to the vent; and the number of postcloacal tubercles (**PCT**). It should be noted that the banding pattern within in *Cyrtodactylusconsobrinus* sensu lato is essentially reversed from that of most other *Cyrtodactylus.* The thin white “bands” are homologous with the pale-colored interspaces of most other species and the dark “interspaces” are homologous with the dark bands of most other species. Color pattern meristics taken were the number of dark body bands (**BB**) between the nuchal loop (the dark band running from eye to eye across the nape) if present and the hind limb insertions; pale and dark markings on the tail are referred to as caudal bands and counted as such; number of dark-colored (**DCB**) caudal bands; and the number of pale-colored caudal bands (**LCB**). Museum acronyms follow Sabaj (2023) except for LSUDPC La Sierra University Digital Photo Collection, Riverside, California, USA.

### ﻿Statistical analyses

All statistical analyses were conducted using R Core Team (2024). A Levene’s test for meristic and size-corrected morphometric characters was conducted to test for equal variances across all groups. To remove potential effects of allometry in the morphometric characters (see Chan and Grismer 2022), size was adjusted using the following equation: X_adj_ = log(X)-β[log(SVL)-log(SVL_mean_)], where X_adj_ = adjusted value; X = measured value; β = unstandardized regression coefficient for each population; and SVL_mean_ = overall average SVL of all populations (Thorpe 1975, 1983; Turan 1999; Lleonart et al. 2000). The statistical analyses employed on all data sets included combinations of multiple factor analysis (MFA) from the R package *FactorMineR* (Husson et al. 2017) and visualized using the *Factoextra* package (Kassambara and Mundt 2017); principle component analysis (PCA) and discriminant analysis of principle components (DAPC) from the *adegent* package 2.1.5 in R (Jombart 2022; PCAtest from Camargo (2022); non-parametric permutation multivariate analysis of variance (PERMANOVA) from the *vegan* package 2.5-3 in R (Oksanen et al. 2020); and analysis of variance (ANOVA) followed by a TukeyHSD *post hoc* test.

#### ﻿*Cyrtodactylusmalayanus* group

To statistically quantify morphological differences among the *malayanus* group species, it was necessary to use the limited data sets of Davis et al. (2020, 2023) augmented with 52 specimens of peninsular *Cyrtodactylusconsobrinus* (Suppl. materials 1, 2). Limited dataset 1 (Suppl. material 1) utilized only four adjusted (see below) morphometric (SVL, HL, HW, HD) and five meristic characters (SL, IL, PVT, LVS, T4TL) but included all *malayanus* group species. Expanded dataset 2 (Suppl. material 2) utilized 10 adjusted morphometric (SVL, HL, HW, HD, ForeL, BracL, TibL, FemL, AG, ES) and nine meristic characters (SL, ILPVT, LRT, LVS, E4TL, U4TL, T4LT, PS) but excluded *C.malayanus*, *C.kapitensis*, and *C.limajalur* for which complete data were unavailable. Having more than twice the number of characters, dataset 2 was more focused on differences among peninsular *C.consobrinus* and its closest Bornean relatives *C.consobrinus* and *C.hutan*. Each data set was subjected to MFA, PERMANOVA, ANOVA, and TukeyHSD analyses.

#### ﻿Peninsular populations

An MFA analysis of the six peninsular lineages of *Cyrtodactylusconsobrinus* (see below) based on 52 individuals was employed to search for (dis)similarities among them in morphospace. The MFA used a concatenated dataset consisting of 16 adjusted morphometric (SVL, BracL, ForeL, FemL, TibL, AG, HL, HW, HD, ED, EE, SN, EN, IO, EarL, and IN) and 14 meristic (SL, IL, PVT, LRT, LVS, TVS, FS, PS, PPS, E4TL, U4TL, T4TL, INA and BB) characters (Suppl. material 3). In an MFA, each individual is described by a different set of variables (i.e., characters) which are structured into different data groups in a global data frame, in this case, quantitative data (i.e., meristic and size-adjusted morphometrics). The different datasets are standardized within the analysis so that one dataset cannot overleverage another (Pagès 2015; Kassambara and Mundt 2017). The plots were evaluated to assess how well they aligned with the mitochondrial lineages delimited in phylogenetic analyses. An ANOVA was employed to search for significant differences in character means among the populations.

To determine which data type contributed most to the variation among the populations, body shape (i.e., morphometric) or meristics, PCAs were employed. Unlike MFA, a PCA does not standardize different data types in the same data frame so separate PCAs and DAPCs were run for the adjusted morphometrics and meristics. Prior to running the DAPCs, PCAtest (Carmago 2022) was employed to reduce the dimensions of the data and recover the significant signal within the PCA data so as to determine how many PCs to retain for the DAPC. The two data frames are combined as separate character types in Suppl. material 3.

PERMANOVA analyses were employed on all MFA and PCA data to determine if there were statistical differences in centroid locations and group clusters among each species/lineage (Skalski et al. 2018). The analysis calculates a Euclidean (dis)similarity matrix using 50,000 permutations. A *p*-value < 0.05 signifies a statistically significant difference between species/lineage pairs. PERMANOVA provides a statistically defensible method of concluding which species/lineage plots are significantly different from others in contrast to the subjective *ad hoc* “eyebaling it” method as is usually done in multivariate plots.

### ﻿Species delimitation

The general lineage concept (GLC: de Queiroz 2007) adopted herein proposes that a species constitutes a metapopulation of organisms evolving independently from other such populations usually owing to a lack of gene flow. By “independently,” it is meant that new mutations arising in one species cannot spread readily into another species (Barraclough et al. 2003; de Queiroz 2007). Under the GLC implemented herein, molecular phylogenies were used to recover monophyletic mitochondrial lineages of individual(s) (i.e., populations) in order to develop initial species-level hypotheses, the grouping stage of Hillis (2019). Univariate, multivariate, and discrete color pattern and morphological data were used to search for statistically significant unique characters and patterns and compare their consistency with the previous species-level hypotheses designations—the construction of boundaries representing the hypothesis-testing step of Hillis (2019)—thus providing lineage diagnoses independent of the molecular analyses. In this way, the inherent errors of simultaneously delimiting (phylogeny) and diagnosing (taxonomy) species are avoided (Frost and Hillis 1990; Frost and Kluge 1994; Hillis 2019).

## ﻿Results

The ML and BI analyses recovered well-supported trees with concordant topologies (Fig. 1). Similar to Davis et al. (2023), the node subtending *Cyrtodactylushutan* and peninsular and Bornean populations of *C.consobrinus* is strongly supported (UFB 100/BPP 1.00). However, the sister group relationship of *C.hutan* and Bornean *C.consobrinus* is not supported (70/0.00) herein. In Davis et al. (2023), Bornean *C.consobrinus* is the poorly supported (90/0.86) sister lineage to peninsular *C.consobrinus*. Importantly however, in both analyses all three populations were monophyletic (100/1.00 herein)—that is no individuals are nested within the population of other individuals—even though their relationships remain unresolved.

**Figure 2.**
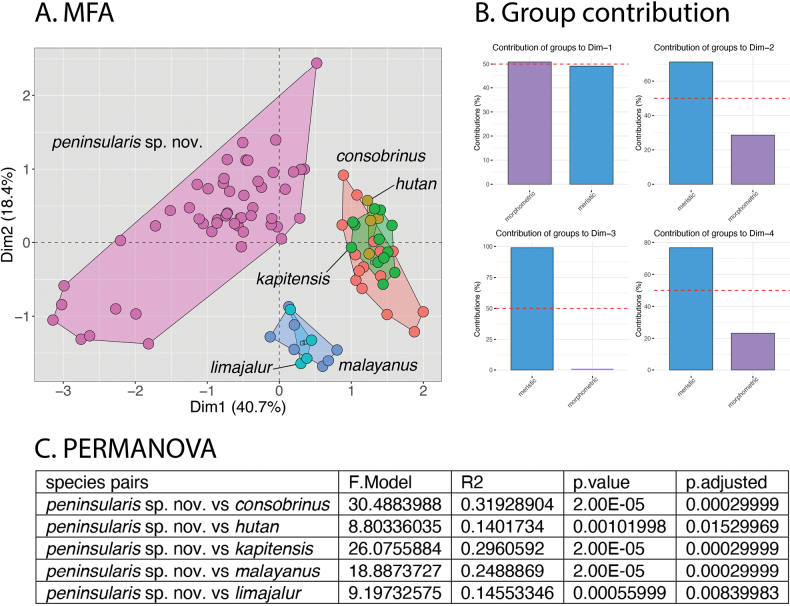
**A** MFA based on four morphometric and five meristic characters comparing all species in the *malayanus* group **B** percent contibution of the morphometric and meristic characters to the overall variation in Dims 1–4 **C** results of the PERMANOVA showing that *Cyrtodactyluspeninsularis* sp. nov. plots significantly different from all other species in morphospace.

In MFA of the *malayanus* group based on Dataset 1, peninsular *C.consobrinus* is statistically well separated from all species (Fig. 2A, C). *Cyrtodactylusmalayanus* and *C.limajalur* overlap completely as do Bornean *C.consobrinus*, *C.hutan*, and *C.kapitensis*. Dimension (Dim) 1 accounts for 40.7% of the variation with the morphometric and meristic data contributing nearly equally (50%) to the 40.7% (Fig. 2B). Dimension 2 accounts for 18.4% of the variation with the meristic data contributing ~ 70% of the variation and the morphometric data ~ 3 0% of the variation of the total 18.4% of Dim 2 (Fig. 2B). The ANOVA demonstrated that peninsular *C.consobrinus* differs significantly from all Bornean members of the *C.malayanus* group in varying combinations of species across varying combinations of characters (Table 2).

**Table 2.** Statistically significant differences (p < 0.05) of means of morphometric and meristic characters between *Cyrtodactyluspeninsularis* sp. nov. and other species in the *malayanus* group based on the ANOVA. ns = no significance. Abbreviations are in the Materials and methods.

	*C.consobrinus*	*C.hutan*	*C.kapitensis*	*C.limajalur*	*C.malayanus*
**HD**	0.00208662	ns	0.00085438	ns	ns
**HL**	0.01454803	ns	ns	ns	ns
**HW**	0.02668742	ns	0.00272659	ns	ns
**SVL**	0.02019303	ns	ns	ns	ns
**SL**	3.71E-10	0.01744459	0.00548214	5.46E-06	0.00236984
**IL**	ns	0.0139327	3.89E-10	0.00018719	5.88E-05
**PVT**	3.56E-10	3.56E-10	3.56E-10	8.50E-05	3.56E-10
**LVS**	4.63E-05	0.00338301	1.93E-05	ns	3.56E-10
**TL4T**	ns	ns	ns	0.00255717	ns

**Figure 3.**
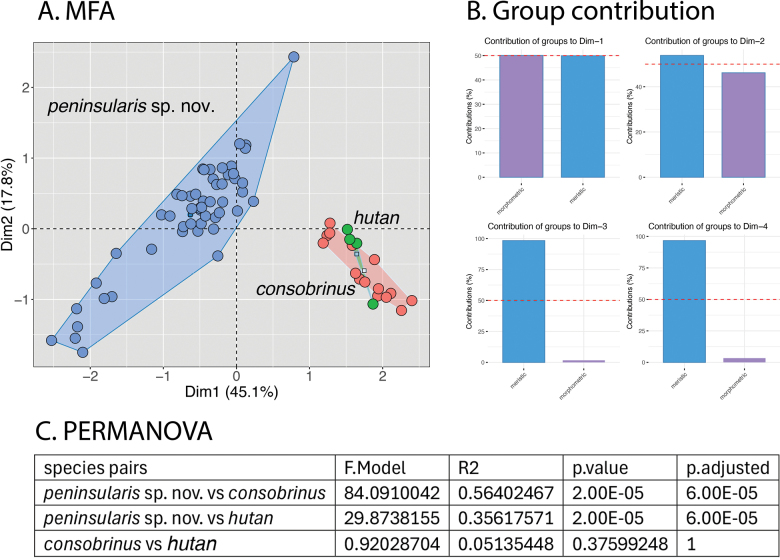
**A** an expanded MFA based on 10 morphometric and nine meristic characters *Cyrtodactyluspeninsularis* sp. nov. and its two closest relatives *C.consobrinus* and *C.hutan***B** percent contirbution of the morphometric and meristic characters to the overall variation in Dims 1–4 **C** results of the PERMANOVA showing that *Cyrtodactyluspeninsularis* sp. nov. plots significantly different from *C.consobrinus* and *C.hutan*.

The MFA of the *malayanus* group based on Dataset 2 mirrored the previous MFA in that peninsular *C.consobrinus* is statistically well separated from Bornean *C.consobrinus* and *C.hutan* although the latter two species completely overlap and are not significantly different from one another (Fig. 3A, C). Dimension 1 accounted for 45.1% of the variation with the morphometric and meristic data contributing equally (50%) to the 45.1% (Fig. 3B). The meristic data contributed to ~ 53% of the variation and the morphometric data ~ 45% of the variation of the total 17.8% of Dim 2 (Fig. 3B). The ANOVA demonstrated that peninsular *C.consobrinus* differs significantly from Bornean *C.consobrinus* and *C.hutan* in AG, SN, FemL, ForeL, HD, HL, HW, SVL, and TibL (Table 3). In meristics, it differs significantly from them in SL, IL, LRT, PVT, LVS and PS (Table 3). However, Bornean *C.consobrinus* and *C.hutan* show now no significant differences from one another in either data set. Peninsular *C.consobrinus* has an uncorrected pairwise sequence divergence of 4.5% and 7.2% from its closest relatives Bornean *C.consobrinus* and *C.hutan*, respectively.

**Table 3.** Statistically significant differences (p < 0.05) based on the ANOVA of meristic and morphometric character means between *Cyrtodactyluspeninsularis* sp. nov. and *C.consobrinus* and *C.hutan.* There were no statistically significant differences between any means of the latter two species. Abbreviations are in the Materials and methods.

	*C.consobrinus*	*C.hutan*		*C.consobrinus*	*C.hutan*
**morphometric data**			**meristic data**		
**AG**			**IL**		
*Cyrtodactyluspeninsularis* sp. nov.	0.00048417	0.03845923	*Cyrtodactyluspeninsularis* sp. nov.	2.44E-10	0.00291076
**SN**			**LRT**		
*Cyrtodactyluspeninsularis* sp. nov.	0	1.37E-08	*Cyrtodactyluspeninsularis* sp. nov.	7.38E-07	0.00202505
**FemL**			**LVS**		
*Cyrtodactyluspeninsularis* sp. nov.	0.00088311	ns	*Cyrtodactyluspeninsularis* sp. nov.	3.27E-05	0.0015292
**ForeL**			**PS**		
*Cyrtodactyluspeninsularis* sp. nov.	7.27E-06	0.00505335	*Cyrtodactyluspeninsularis* sp. nov.	0.00102883	7.82E-05
**HD**			**PVT**		
*Cyrtodactyluspeninsularis* sp. nov.	0.00216919	ns	*Cyrtodactyluspeninsularis* sp. nov.	0	0
**HL**			**SL**		
*Cyrtodactyluspeninsularis* sp. nov.	0.01114378	0.04149057	*Cyrtodactyluspeninsularis* sp. nov.	0	0.00427914
**HW**					
*Cyrtodactyluspeninsularis* sp. nov.	0.01807166	0.0465810			
**SVL**					
*Cyrtodactyluspeninsularis* sp. nov.	0.01523904	ns			
**TibL**					
*Cyrtodactyluspeninsularis* sp. nov.	7.42E-08	0.00171716			

The phylogenetic relationships within peninsular *C.consobrinus* are geographically well-structured and comprised of six strongly supported monophyletic lineages associated with particular geographic regions (Fig. 1): a northwestern lineage (NWL) in hilly areas of north and northwestern Peninsular Malaysia and extreme southern Thailand; a northeastern lineage (NEL) in the northern section of the easternmost ranges of the Banjaran Timur in northeastern Peninsular Malaysia; a north-central lineage (NCL) from lowland and upland areas across the northeastern arm of the Banjaran Titiwangsa and the Banjaran Timur in northern Peninsular Malaysia; a widespread eastern lineage (EL) ranging in hilly regions of the southeastern most Banjaran Timur in the north to Endau-Rompin in the south; a western lineage (WL) ranging along the Banjaran Titiwangsa from at least Gunung Korbu in the north to the isolated Gunung Ledang in the south; and a southern lineage (SL) centered around the hilly regions of Gunung Belumut, Gunung Panti, and Gunung Pulai. Owing to their monophylies, there is currently no evidence of mitochondrial gene flow among any of the lineages and in fact, LSUHC 11201 from Puncak Air on Gunung Tebu in the north-central lineage (#7) was found at 610 m in elevation and LSUHC 10879 from the base of Gunung Tebu at Lata Belatan (#1) in the eastern lineage found at 42 m in elevation, are separated by only ca 568 m in elevation (Sumarli et al. 2015).

**Figure 4.**
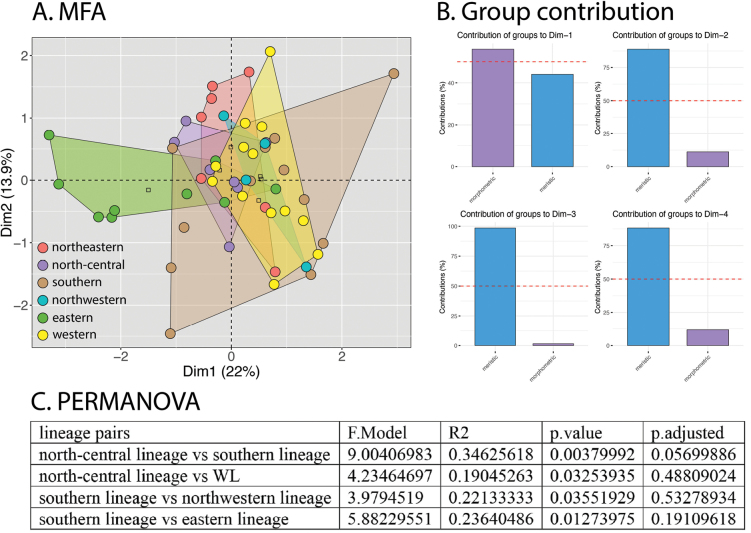
**A** MFA of the lineages of *Cyrtodactyluspeninsularis* sp. nov. based on 15 morphometric and 16 meristic characters **B** percent distribution of the morphometric and meristic characters to the overall variation in Dims 1–4 **C** results of the PERMANOVA showing lineage pairs that differed significantly from each other.

The MFA of the lineages of the peninsular *Cyrtodactylusconsobrinus* show a great degree of overlap with only four pairs of lineages being statistically different from one another (Fig. 4A, C). Dimension 1 accounted for 22.0% of the variation with the morphometric data contributing ~ 57% and meristic data ~ 45% of the 22% variation (Fig. 4B). The meristic data contributed to ~ 90% and the morphometric data ~ 12% of the total 13.9% of the variation in Dim 2 (Fig. 4B).

**Figure 5.**
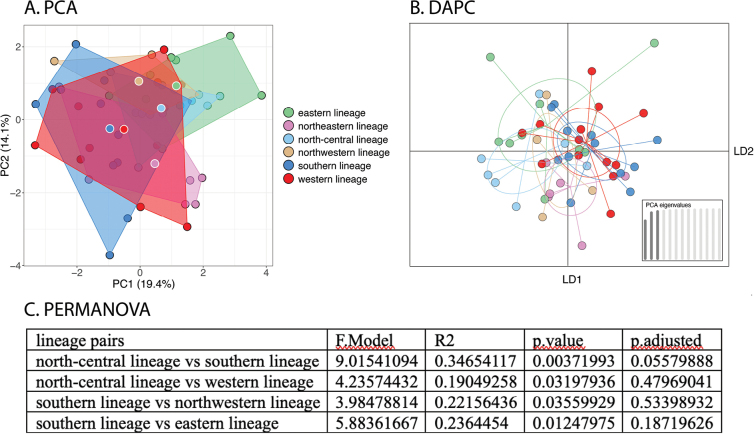
**A**PCA of the lineages of *Cyrtodactyluspeninsularis* sp. nov. based on 16 meristic characters **B**DAPC of the same data set based on the retention of the first three PCs **C** results of the PERMANOVA showing the lineages of *C.peninsularis* sp. nov. that plot significantly different from each other.

**Figure 6.**
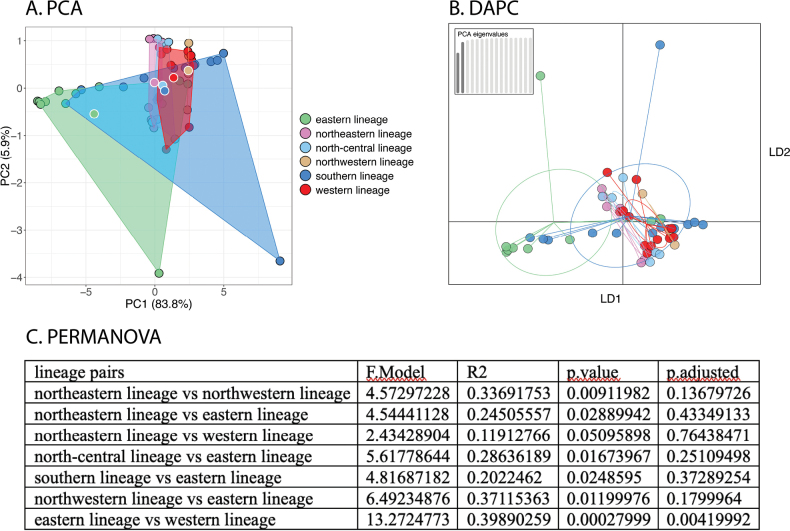
**A**PCA of the lineages of *Cyrtodactyluspeninsularis* sp. nov. based on 15 morphometric characters **B**DAPC of the same data set based on the retention of the first two PCs **C** results of the PERMANOVA showing the lineages of *C.peninsularis* sp. nov. that plot significantly different from each other. The eastern lineage differs from all others.

PCAs and DAPCs for the lineages of peninsular *Cyrtodactylusconsobrinus* were conducted separately on the morphometric and meristic data sets in order to discern the contribution of each to differences of body shape and meristics to the overall variation among the lineages. Despite the well-supported phylogeographic structure, only limited morphospatial differences among the lineages were recovered as evidenced by the considerable overlap among all lineages in the PCAs and DAPCs (Figs 5, 6). Even so, various lineages plotted significantly differently from other lineages but with no apparent geographical concordance (e.g., geographically distant lineages such as NWL and SL were not necessarily significantly different [Fig. 5C] whereas the converse was sometimes true as in SL and EL, and NWL and EL [Figs 5C, 6C, respectively]). Summary statistics for both PCAs are in Suppl. material 4. The ANOVA of the adjusted morphometric characters showed the most variation and recovered the eastern lineage as distinct from all other lineages in a several characters whereas there were no significant morphometric differences among the other lineages (Table 4). The ANOVA of the meristic characters recovered the eastern lineage as significantly different from all other lineages only in the mean number of infralabial scales and from the southern population in the number of transverse ventral scales (Table 5). Only four other significant differences among other lineages were recovered. The uncorrected pairwise sequence divergence among the six lineages ranged from 0.97% between NEL and NCL to 4.5% between SL and NCL (Table 6).

**Table 4.** Statistically significant differences (p < 0.05) among the means of adjusted morphometric characters between the eastern lineage and the other five lineages of *Cyrtodactyluspeninsularis* sp. nov. based on the ANOVA. Abbreviations are in Materials and methods.

AG	eastern	ForeL	eastern	EE	eastern	IN	eastern
northeastern		northeastern		northeastern	0.03833241	northeastern	
northcentral		northcentral		northcentral		northcentral	0.04698467
southern		southern	0.02441878	southern	0.00988557	southern	0.00568237
northwestern		northwestern	0.04406496	northwestern	0.01771997	northwestern	0.01143519
eastern		eastern		eastern		eastern	
western	0.017352981	western	0.0086528	western	0.00138853	western	0.00115093
**BracL**		**HD**		**EN**		**IO**	
northeastern		northeastern		northeastern		northeastern	0.03322538
northcentral		northcentral	0.02137127	northcentral	0.0548663	northcentral	0.00419788
southern	0.025259163	southern	0.01295353	southern	0.01516702	southern	0.01003158
northwestern		northwestern	0.00730988	northwestern	0.01090641	northwestern	0.00082774
eastern		eastern		eastern		eastern	
western	0.005804612	western	0.00299493	western	0.00487811	western	0.00047025
**EarL**		**HL**		**FemL**		**SN**	
northeastern	0.017276883	northeastern		northeastern		northeastern	
northcentral		northcentral	0.05060414	northcentral	0.03824613	northcentral	0.04561404
southern	0.001794086	southern	0.00849435	southern	0.02032371	southern	0.01249032
northwestern	0.015886527	northwestern	0.01220355	northwestern	0.03684145	northwestern	0.00848437
eastern		eastern		eastern		eastern	
western	0.009340077	western	0.00170115	western	0.00485775	western	0.00214369
**ED**		**HW**		**TibL**			
northeastern		northeastern	0.03879641	northeastern			
northcentral		northcentral	0.01550305	northcentral			
southern	0.03106832	southern	0.00421885	southern			
northwestern	0.023975227	northwestern	0.00429371	northwestern			
eastern		eastern		eastern			
western	0.037119934	western	0.00105094	western	0.01185257		

**Table 5.** Statistically significant differences (p < 0.05) among the means of meristic characters among lineage combinations of *Cyrtodactyluspeninsularis* sp. nov. based on the ANOVA analyses. ns = no significance. Abbreviations are in Materials and methods.

FS	northcentral	northwestern	eastern	PVT	northcentral	eastern
northeastern	0.047778828	ns	ns	northeastern	ns	ns
northwestern	ns	ns	ns	northwestern	0.01550179	ns
eastern	ns	ns	ns	eastern	ns	ns
**IL**				**TVS**		
northeastern	ns	ns	0.018419858	northeastern	ns	ns
northcentral	ns	ns	0.001204268	northcentral	ns	ns
southern	ns	ns	0.046936213	southern	ns	0.04495103
northwestern	ns	ns	0.005434208	northwestern	ns	ns
**INA**						
northeastern	ns	0.027522026				
**PVT**						
northwestern	0.01550179	ns	ns			
**TVS**						
southern	ns	ns	0.04495103			

**Table 6.** Uncorrected pairwise sequence divergence among the lineages of *Cyrtodactyluspeninsularis* sp. nov. based on a 1459 base pair gene fragment of ND2 and its flanking tRNAs. Other abbreviations are in Materials and methods.

	NEL	NCL	SL	NWL	EL	WL
NEL						
NCL	0.0097					
SL	0.0372	0.0451				
NWL	0.0342	0.0382	0.0363			
EL	0.0355	0.0346	0.0338	0.0216		
WL	0.0337	0.0355	0.0278	0.0208	0.0106	

### ﻿Taxonomy

The phylogenetic relationships within the *malayanus* group indicate that peninsular *Cyrtodactylusconsobrinus* and Bornean *C.consobrinus* form distinct monophyletic mitochondrial lineages separated by a sequence divergence of 4.5% (Fig. 1). The PERMANOVAs of the MFAs show that peninsular *C.consobrinus* plots significantly different from all other species in the *malayanus* group (Figs 2C, 3C) and the ANOVAs recovered peninsular *C.consobrinus* as significantly different from Bornean *C.consobrinus* in nine morphometric and six meristic characters (Tables 2, 3). Based on these data and their allopatry, clearly precluding any possibility of contemporary gene flow, we consider peninsular *C.consobrinus* to be an unnamed species that we describe below.

The well-supported phylogeographic structure within peninsular *C.consobrinus* is discordant with the morphospatial similarity among its lineages, nor is it strongly corroborated in the ANOVA analyses, save for the eastern lineage. Thus, at this juncture, we consider peninsular *C.consobrinus* to be a single new species. Given that these vagile geckos are common inhabitants of both primary and secondary lowland and upland forests (Grismer 2011), the possibility of admixture among the lineages will remain unresolved in the absence of a genomic dataset. Therefore, we elect not to recognize any of the lineages as distinct species. *Cyrtodactylusconsobrinus* sensu stricto is restricted to Borneo given that the type locality is “Sarawak, [Borneo]” (Peters 1871: 569).

#### ﻿ Cyrtodactylus peninsularis sp. nov.

Taxon classificationAnimaliaSquamataGekkonidae

1F122185-B6AF-54EF-B000-ACD2B20EC3D7

https://zoobank.org/AEE782A0-E37B-4815-88E0-9768947965EA

Figs 7 , 8 , 9

Gymnodactylus consobrinus — Boulenger 1912: 37 (part); Smith 1930: 13 (part); Bourret 2009: 123 (part).
Cyrtodactylus consobrinus — Grandison 1972: 81 (part); Dring 1979: 181 (part); Denzer and Manthey 1991: 314 (part); Kluge 2001: 8 (part); Cox et al. 1998: 88 (part); Chan-ard et al. 1999: 1058, 1064–65 (part), 2015: 49, 51 (part); Grismer 2008: 30 (part), 2011: 386 (part); Rösler 2016: 12 (part); Grismer and Quah 2019: 233; Sumarli et al. 2015: 5, 19 (part); Hong et al. 2021:799, 800 & 807; Quah et al. 2021:241 & 246; Davis et al. 2023: 3 (part); Poyarkov et al. 2023: 299, 301 (part).
Cyrtodactylus consubrinus — Manthey and Grossmann 1997: 221 (part; unjustified subsequent spelling).
Cyrtodactylus cf. consobrinus — Figueroa et al. 2023: 100.
Cyrtodactylus (Cyrtodactylus) consobrinus — Rösler 2000: 65 (part).

##### Type material.

***Holotype*** • Adult male (LSUHC 10202) collected from the base of Gunung Belumut, Johor State, Peninsular Malaysia (2.065817°N, 103.526119°E at 245 m) on 8 September 2009 by Evan S. H. Quah, Perry L. Wood, Jr., Jesse L. Grismer, L. Lee Grismer, Shahrul Anuar, and Mohamed A. Muin. ***Paratypes*.** • Adult females: LSUHC 9333 northwestern lineage collected from Sungai Sedim, Kedah State, Peninsular Malaysia (5.414063°N, 100.779803°E at 129 m) on 16 March 2009; LSUHC 10048 northeastern lineage collected from Hutan Lipur Sekayu, Terengganu State, Peninsular Malaysia (4.980644°N, 102.934645°E at 441 m) on 27 March 2009; LSUHC 11136–37 north-central lineage collected from Gunung Stong, Kelantan State, Peninsular Malaysia (5.321880°N, 101.964944°E at 703 m) on 27 June 2009; LSUHC 11152 north-central lineage collected from Hutan Lipur Jelawang, Kelantan State, Peninsular Malaysia (5.340351°N, 101.969544°E at 699 m) on 27 June 2009; LSUHC 11979 northeastern lineage collected from Sungai Bubu, Terengganu State, Peninsular Malaysia (5.0120261°N, 102.952963°E at 77 m) on 1 September 2009; and LSUHC 12386 eastern lineage collected from Hutan Lipur Chemerong, Terengganu State, Peninsular Malaysia (4.660664°N, 103.001320°E at 129 m) on 17 August 2009. Adult males: LSUHC 10230 southern lineage collected from the base of Gunung Belumut, Johor State, Peninsular Malaysia (2.045596°N, 103.530185°E at 244 m) on 9 September 2009 by Evan S. H. Quah, Perry L. Wood, Jr., Jesse L. Grismer, L. Lee Grismer, Shahrul Anuar, and Mohamed A. Muin; and LSUHC 11267 northwestern lineage collected from trail 2, Sungai Enam, Belum, Perak State, Peninsular Malaysia (5.46768°N,101.28961°E on 6 October 2009.

##### Additional specimens examine

**(*n* = 52).** See Suppl. material 5.

##### Diagnosis based on type series.

*Cyrtodactyluspeninsularis* sp. nov. can be separated from all other species of the *malayanus* group by the combination of having a maximum SVL of 128.7 mm (female); 8–10 supralabials; 10–12 infralabials; 25–30 paravertebral tubercles; 15–20 rows of longitudinally arranged tubercles; 40–62 longitudinal rows of ventrals; 243–299 transverse rows of ventrals; 7–9 expanded subdigital lamellae on the fourth toe; 13–16 unmodified subdigital lamellae on the fourth toe; 21–25 total subdigital lamellae on the fourth toe; 21–25 total number of enlarged femorals; 2–9 total number of femoral pores in males, no femoral pores in females; 10–12 enlarged precloacals; nine or ten precloacal pores in males (*n* = 3), precloacal pores in some females (three of seven); two or three rows of large post-precloacals; two postcloacal tubercles (spines) on each side; dorsal pattern extremely variable, dark dorsal bands very wide reducing the pale dorsal interspaces to 2–4 thin lines; seven or eight dark and pale caudal bands (*n* = 3); large moderately keeled body tubercles; caudal tubercles extend beyond base of tail; subcaudals transversely expanded but not extending high up onto side of tail; enlarged distal femorals and enlarged precloacals not contiguous; no enlarged proximal femorals; top of head overlain with reticulating white network of thin lines; dark caudal bands wider than pale caudal bands; dark markings usually within pale caudal bands in adults (Tables 7, 8, Figs 5, 6, Suppl. material 2).

**Table 7.** Meristic and raw morphometric characters of the type series of *Cyrtodactyluspeninsularis* sp. nov. na = data unavailable, R = regenerated.

Catalog number	LSUHC 10202	LSUHC 09633	LSUHC 10048	LSUHC 10230	LSUHC 11136	LSUHC 11137	LSUHC 11152	LSUHC 11267	LSUHC 11979	LSUHC 12386
	holotype	paratype	paratype	paratype	paratype	paratype	paratype	paratype	paratype	paratype
sex	M	F	F	M	F	F	F	M	F	F
**Meristic characters**										
supralabials (SL)	9	8	8	8	9	10	9	9	9	10
infralabials (IL)	11	12	11	11	11	11	10	10	11	11
internasals (INA)	3	2	4	5	3	3	2	2	4	2
paravertebral tubercles (PVT)	30	30	28	29	25	25	25	29	29	29
longitudinal tubercle rows (LRT)	16	17	18	20	16	16	15	19	17	17
longitudinal ventral scale rows (LVS)	47	41	62	56	40	54	40	41	52	56
transverse ventral scale rows (TVS)	252	265	281	251	279	279	274	269	243	299
total enlarged femoral scales (FS)	12	12	12	13	10	12	na	12	12	11
femoral pores-right	4	na	na	5	na	na	na	2	na	na
femoral pores-left	3	na	na	4	na	na	na	3	na	na
total femoral pores (FP)	7	na	na	9	na	na	na	5	na	na
enlarged precloacals (PS)	12	10	11	12	12	12	10	11	10	11
precloacal pores (PP)	9	na	10	10	10	na	na	9	na	10
post-precloacal scales (PPS)	3	2	3	2	3	2	3	3	3	3
postcloacal tubercles (CT)	2	2	2	2	2	2	2	2	2	2
expanded 4^th^ toe lamellae (E4TL)	7	8	9	8	8	9	9	8	8	8
unexpanded 4^th^ toe lamellae (U4TL)	16	16	16	16	16	14	16	13	14	16
total 4^th^ toe lamellae (T4TL)	23	24	25	24	24	23	25	21	22	24
dark body bands (BB)	3	2	3	4	3	3	3	2	3	2
dark colored caudal bands (DCB)	7	6R	na	B	8	R	8	na	B	B
pale colored body bands (LCB)	7	5R	na	B	7	R	8	na	B	B
**Morphometric characters (mm)**										
snout-vent length (SVL)	113.10	121.1	115.6	112.4	121.9	128.7	126.2	125.0	122.6	112.0
tail length (TL)	136.8	136.7	120.0	131.0R	132.45	108.8	139.9	118.1R	116.8R	86.6R
tail width (TW)	8.6	7.7	10.0	8.3	7.6	9.4	8.3	8.9	8.1	8.5
brachial length (BracL)	19.0	19.9	19.6	19.2	21.2	19.8	21.0	21.0	18.2	18.2
forearm length (ForeL)	17.6	18.8	18.0	18.2	20.0	19.2	20.4	17.34	17.9	18.8
femoral length (FemL)	22.7	23.0	22.1	23.1	25.7	25.2	24.8	23.1	22.8	20.7
tibia length (TibL)	20.2	19.3	20.9	19.4	20.6	22.2	21.8	18.9	20.2	19.7
axilla-groin length (AG)	52.6	54.2	49.7	54.6	53.2	58.9	54.8	58.2	51.7	52.4
head length (HL)	33.1	35.4	32.7	33.1	33.8	35.6	35.1	35.4	34.5	31.8
head width (HW)	20.4	21.9	21.7	21.7	22.6	24.8	23.6	24.5	22.7	20.4
head depth (HD)	12.3	12.1	12.0	12.8	13.5	13.9	13.7	13.9	12.2	11.8
eye diameter (ED)	7.0	7.4	6.9	7.0	7.3	8.1	7.5	7.1	7.3	7.0
eye to ear distance (EE)	7.4	8.9	9.1	8.8	9.3	7.5	10.5	9.1	9.4	8.2
snout length (SN)	12.7	13.4	12.9	12.4	13.7	14.6	14.5	13.7	13.8	12.3
eye to nostril distance (EN)	9.8	10.1	10.3	10.0	10.7	9.1	11.5	10.7	10.8	9.6
interorbital distance (IO)	10.1	9.0	10.7	10.8	11.6	12.2	12.34	12.1	9.4	9.5
ear length (EarL)	2.2	2.7	2.6	1.8	1.6	1.5	1.6	1.9	1.8	1.9
internarial distance (IN)	3.8	3.6	3.6	3.9	3.9	5.0	4.8	4.2	4.3	3.6

**Table 8.** Summary statistics of the meristic characters *Cyrtodactyluspeninsularis* sp. nov. Abbreviations are in Materials and methods.

	SL	IL	PVT	LRT	LVS	TVS	FS	PS	PPS	E4TL	U4TL	T4TL	INA	BB	FP	PP	LCB	DCB
mean	8.9	10.9	27.9	17.1	48.9	269.2	11.8	11.1	2.7	8.2	15.3	23.5	3	2.7	7.0	9.7	7.7	7.3
±sd	0.74	0.57	2.08	1.52	8.13	16.91	0.79	0.88	0.48	0.63	1.16	1.27	1.05	0.67	2.99	0.52	0.58	0.58
range	8–10	10–12	25–30	15–20	40–62	243–299	10–13	10–12	2 or 3	7–9	13–16	21–25	2–5	2–4	2–9	9 or 10	7 or 8	7 or 8
N	10	10	10	10	10	10	10	10	10	10	10	10	10	10	3	6	3	3

##### Description of holotype

**Figure 7.**
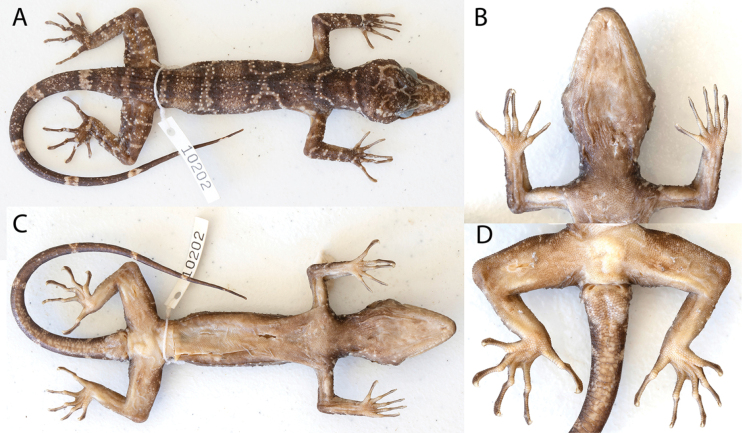
**A, C** dorsal and ventral views of adult male holotype of *Cyrtodactyluspeninsularis* sp. nov. LSUDPC 10202, respectively **B** gular region, throat, pectoral region, and ventral view of forelimbs **D** precloacal region, ventral view of hind limbs, and anterior subcaudal region.

**(Fig. 7, Table 7).** Adult male SVL 113.1 mm; head moderate in length (HL/SVL 0.29), width (HW/HL 0.62), somewhat flattened (HD/HL 0.37), distinct from neck, triangular in dorsal profile; lores concave anteriorly, inflated posteriorly; prefrontal deeply concave; canthus rostralis rounded; snout elongate (SN/HL 0.38), flat, rounded in dorsal profile; eye large (ED/HL 0.21); ear opening elliptical, obliquely oriented, moderate in size; eye to ear distance slightly greater than diameter of eye; rostral rectangular, partially divided dorsally, bordered posteriorly by large left and right supranasals and slightly smaller internasal, bordered laterally by first supralabials; external nares directed posterolaterally, bordered anteriorly by rostral, dorsally by large supranasal, posteriorly by six small postnasals, ventrally by first supralabial; nine (R,L) rectangular supralabials tapering to below posterior margin of eye, first six supralabials largest; 11 (R,L) infralabials tapering smoothly to slightly past termination of enlarged supralabials; scales of rostrum and lores raised, much larger than granular scales on top of head and occiput; scales of occiput intermixed with small, rounded, tubercles; superciliaries flat, elongate, largest dorsally; mental triangular, bordered laterally by first infralabials and posteriorly by large left and right trapezoidal postmentals contacting medially for ~ 40% of their length posterior to mental; one row of enlarged, sublabials extending posteriorly to fifth infralabials (R,L); gular and throat scales small, granular, grading posteriorly into slightly larger, flatter, smooth, subimbricate pectoral and ventral scales.

Body relatively long (AG/SVL 0.47) with well-defined ventrolateral folds; dorsal scales small, granular, interspersed with large, moderately keeled, semi-regularly arranged tubercles extending from occiput to beyond base of tail; ~ 16 longitudinal rows of tubercles at midbody; ~ 30 paravertebral tubercles; ~ 47 flat, imbricate, ventral scales much larger than dorsal scales; 12 enlarged precloacal scales not separated medially by poreless scales; no deep precloacal groove or depression; and three rows of large post-precloacal scales on midline.

Forelimbs moderate in length and stature (ForeL/SVL 0.16); granular scales of forelimbs slightly larger than those on body, large spinose tubercles on dorsal surface of forearms; palmar scales slightly rounded, juxtaposed; digits well-developed, inflected at basal interphalangeal joints, slightly narrower distal to inflections; subdigital lamellae transversely expanded, those proximal to joint inflections much wider than lamellae distal to inflections; claws well-developed, sheathed by a dorsal and ventral scale; hind limbs robust, wider and longer than forelimbs (TibL/SVL 0.18), covered dorsally by granular scales interspersed with large pointed tubercles; anterior scales of thigh slightly larger and flatter than dorsal scales of thigh; ventral scales of thighs rounded, subimbricate, slightly larger than dorsals; distal subtibials large, flat, subimbricate; one row of six (R, L) distal enlarged femoral scales, four on right bearing pores and three on left bearing pores, no other enlarged femoral scales; proximal femorals not forming an abrupt union with granular posteroventral scales of thigh; plantar scales rounded, juxtaposed; digits well-developed, inflected at basal interphalangeal joints; claws well-developed, sheathed by a dorsal and ventral scale at base; seven (R, L) wide subdigital lamellae on fourth toe proximal to joint inflection, 16 (R, L) narrower lamellae distal to joint inflection, 23 total subdigital lamellae.

Tail long (TL/SVL 1.21), original, tapering to a point; dorsal caudal scales small, generally square, juxtaposed; median row of subcaudals significantly larger than dorsal caudals, transversely expanded, not extending high up dorsally onto lateral side of tail; body tubercles extending beyond base of tail; hemipenial swellings at base of tail, two large postcloacal tubercles on both sides; and postcloacal scales flat, imbricate.

##### Color and pattern in preservative

**(Fig. 7).** No photograph of the living holotype was available. Ground color of top of head, limbs, and dorsum brown; top of head overlain with a reticulating network of thin white lines; snout bearing irregularly shaped white blotches and lines; thin, white, transverse line (i.e., interspace) on occiput and another immediately anterior to shoulders; a thin, white anterior dorsal line bifurcates paravertebrally forming two thin lines along anterior of flanks; a second thin white line occurs just posterior to midway between the limb insertions; another thin white line occurs at level of groin; all white lines bordered by large white tubercles and are thickly edged in dark brown; center of the brown regions between the thin white lines (i.e., body bands) bear irregularly shaped central pale brown areas; a thin white sacral line followed by seven widely spaced white caudal bands bearing darkened markings, separated by dark caudal bands nearly three times width of pale caudal bands; limbs dark brown to pale brown overlain with thin, white broken lines and irregularly shaped markings.

##### Etymology.

The species name *peninsularis* is in reference to the distribution of this species which is restricted to the Thai-Malay Peninsula of southern Thailand, Peninsular Malaysia, and Singapore.

##### Distribution.

*Cyrtodactyluspeninsularis* sp. nov. ranges from extreme southern Thailand southward through nearly all habitats in Peninsular Malaysia to Singapore (Grismer 2011) (Fig. 1). The Pulau Singkep population of Indonesia has not been investigated.

##### Variation.

**Figure 8.**
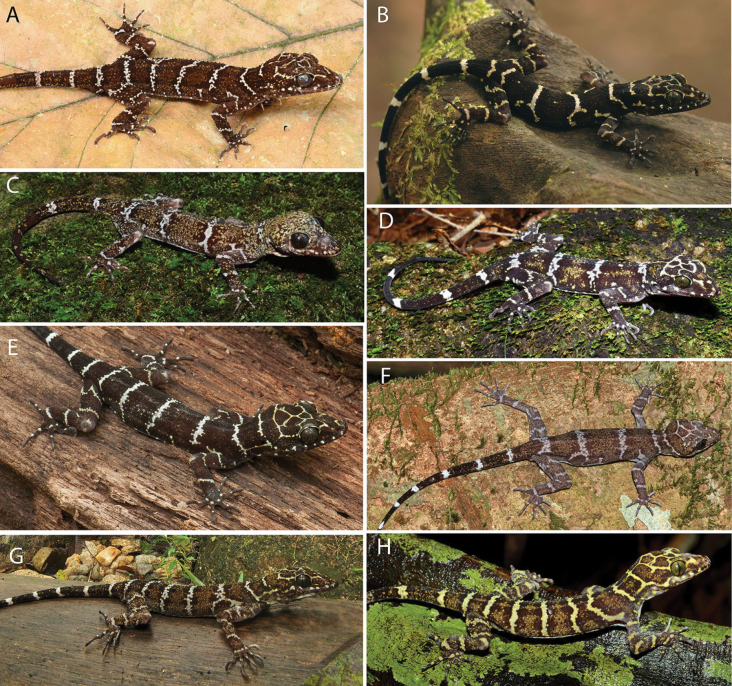
Color and banding pattern variation among the lineages of *Cyrtodactyluspeninsularis* sp. nov. **A**NEL— adult male, Lata Kekabu, Setiu, Terengganu, La Sierra University Digital Photograph Collection (LSUDPC) 13548, photo by Evan S. H. Quah **B**EL—juvenile, Endau-Rompin National Park, Johor, LSUHC 2585, photo by L. Lee Grismer **C**NWL—adult male, Sungai Enam, Perak LSUDPC 13549 (paratype LSUHC 11267), photo by Evan S. H. Quah **D**WL—adult female, Gunung Ledang, Johor LSUDPC 13550, photo by Evan S. H. Quah **E**NCL—adult female, Gunung Tebu, Terengganu LSUDPC 7997, photo by L. Lee Grismer **F**WL—adult female, Gunung Korbu, Perak, LSUDCP 13548, photo by Kin Onn Chan **G**NEL—adult male, Hutan Lipur Sekayu, Terengganu, LSUDPC 5951, photo by L. Lee Grismer **H**SL—adult female, Gunung Pulai, Johor, LSUDPC 13552, photo by Evan S. H. Quah.

**Figure 9.**
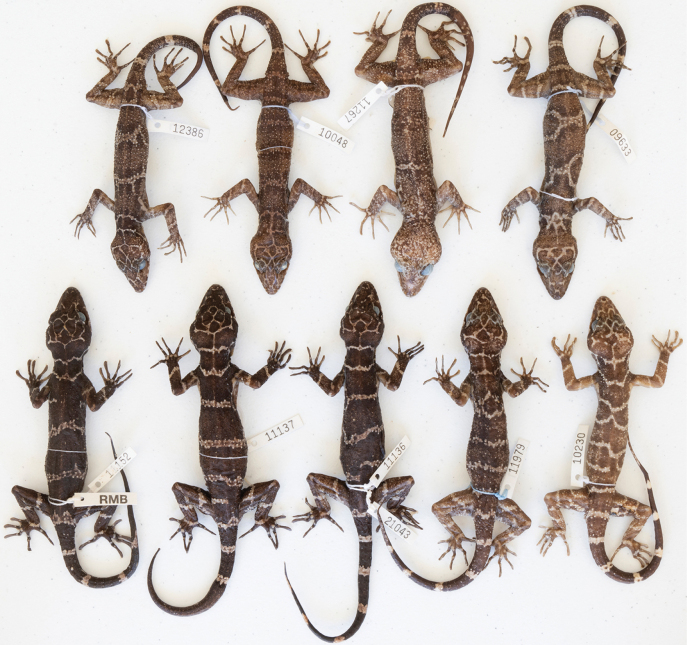
Paratypes of *Cyrtodactyluspeninsularis* sp. nov. showing the wide variation in dorsal banding pattern.

Color pattern varies so extensively within *Cyrtodactyluspeninsularis* sp. nov. it essentially defies a concise meaningful description (e.g., Fig. 8A, C). The type series was chosen to not only cover the geographic range of the species but to cover a great deal of its color pattern variation as well (Fig. 9). This remarkable variation, however, is paradoxically coupled to considerable conservatism in its morphological variation (Figs 4, 5, 6, Tables 4, 5).

##### Comparisons.

**Figure 10.**
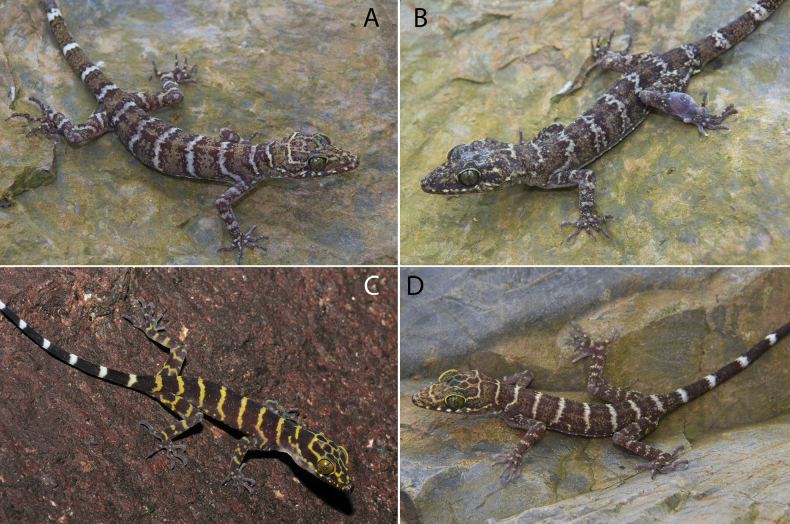
*Cyrtodactylusconsobrinus* from Gua Angin, Sarawak, East Malaysia (Borneo) **A** adult female LSUDPC 10767 **B** adult male LSUDPC 10764 **C** hatchling LSUDPC 4928 **D** Juvenile LSUDPC 10769.

*Cyrtodactyluspeninsularis* sp. nov. may be the sister species of *C.consobrinus* from which it differs morphometrically in having a statistically shorter snout-vent length; axilla-groin length; snout length; tibial and forelimb length; head length, width, and depth, and femur length (Table 3). It differs from *C.consobrinus* meristically by having statistically more infralabials; longitudinal rows of tubercles; enlarged precloacal scales; and fewer supralabial scales, longitudinal ventral scales, and paravertebral tubercles (Table 3). Hatchling *C.peninsularis* sp. nov. nearly always have thin white interspaces whereas they are yellow in *C.consobrinus* (Fig. 10). From the closely related *C.hutan*, *C.peninsularis* sp. nov. differs morphometrically in having a statistically shorter AG, SN, TibL, and ForeL, and shorter HL and HW. It differs meristically in having fewer SL, LVS, PS, and PVT and more IL and LRT (Table 3).

##### Natural history.

**Figure 11.**
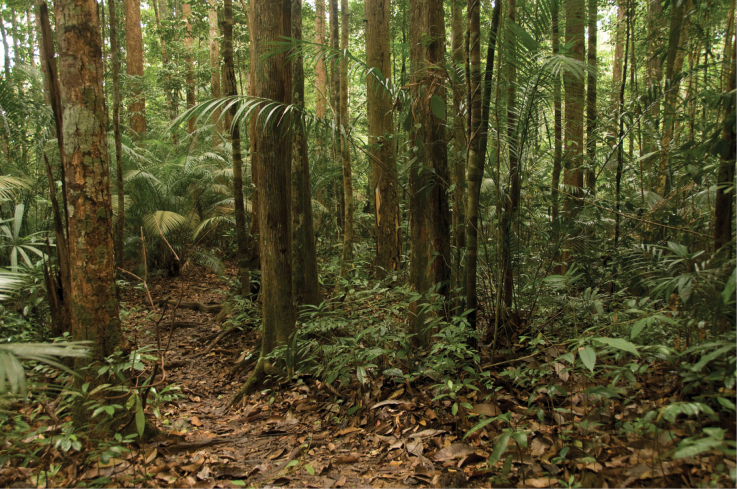
Habitat of the type locality at the base of Gunung Belumut, Johor State.

Much of the following is adapted from Grismer (2011). *Cyrtodactyluspeninsularis* sp. nov. is a vagile, nocturnal, scansorial species that ranges throughout extreme southern Thailand, Peninsular Malaysia, and forested areas of Bukit Timah in Singapore up to ca 800 m in elevation (Chan et al. 2019). *Cyrtodactyluspeninsularis* sp. nov. is a relatively common inhabitant of primary and secondary dipterocarp forests and is occasionally found in peat swamps. Lizards are usually seen climbing on tree trunks, branches, exposed roots, and fallen logs where there are nearby crevices and holes into which they can quickly retreat when threatened. It is not uncommon to find multiple individuals on the same large tree up to 5 m above ground. Lizards are less commonly found among large boulders, taking refuge in cracks or in holes at their base. Anecdotally, this species seems to be more abundant in riparian areas. The holotype was found during the early evening at the base of Gunung Belumut at 245 m in elevation and 2 m above the ground on the trunk of a dipterocarp tree in primary forest (Fig. 11).

## ﻿Discussion

### ﻿Phylogeographic structure versus morphological variation

The discordance between the morphological variation and the geographically structured genetic variation in *Cyrtodactyluspeninsularis* sp. nov. is unlike that seen in other species of *Cyrtodactylus* sharing the same distribution across Peninsular Malaysia. Johnson et al. (2012) constructed an ND2 phylogeny of *C.quadrivirgatus* (*n* = 77), a vagile forest generalist, across its entire range in Peninsular Malaysia and designated 14 monophyletic lineages (“clades” using their rhetoric) from different geographic regions. However, there was little to no support for relationships among the lineages which bore extremely short internode branch lengths and an uncorrected pairwise sequence divergence of only 0.14–3.3%. The distribution of some *C.quadrivirgatus* lineages partially matched that of some of the lineages within *C.peninsularis* sp. nov. but many others showed intraspecific geographic discordance (i.e., closely related lineages were not necessarily geographically close). There was also no discrete morphological differentiation among any of the lineages and a mismatch distribution analysis indicated that *C.quadrivirgatus* is undergoing range expansion.

Grismer et al. (2014) constructed an ND2 phylogeny of the *pulchellus* group species across its range Peninsular Malaysia (*n* = 78) and recovered 11 well supported clades from distinctive geographic regions. These clades were closely aligned to microhabitat preference (i.e., granite, karst, forest, generalist [Grismer et al. 2021]) and bore little interspecific phylogeographic concordance (Grismer et al. 2014: fig. 1) or concordance with the lineages of *C.peninsularis* sp. nov. Each clade was morphologically distinct and given its own species designation. The uncorrected pairwise sequence divergence among the species ranged from 5.5–14.5%.

Surprisingly, there is reasonably strong phylogeographic concordance among peninsular populations of *Amolops*, a fast-flowing rocky stream-adapted ranid frog, and the lineages of *Cyrtodactyluspeninsularis* sp. nov. Chan et al. (2017, 2018) recovered and East 1 clade matching the distribution of the NCL of *C.peninsularis* sp. nov., including a disjunct population on an eastward projecting arm in the northern portion of the Banjaran Titiwangsa. A closely related East 2 clade matched the distribution of the southern portion of the EL and a Larutensis clade matched the distribution of the southwestern arm of the NWL. Lastly, their West 1–4 clades were similar to the WL of *C.peninsularis* sp. nov. and its distribution through the Banjaran Titiwangsa. The East 1 and 2 clades were given their own species designation as *Amolopsgerutu* and *A.australis*, respectively (Chan et al. 2018). These patterns are also congruent with important biogeographic regions inferred by Chan and Grismer (2021) that were based on distribution data from 70 species of frogs and 85 species of lizards. In that study, Chan and Grismer (2021) characterized four significant biogeographic regions: (1) the Eastern Region, which encompasses the distribution of the *A.gerutu* and NCL of *C.peninsularis* sp. nov.; (2) the Southern Region, encompassing the distribution of *A.australis* and EL of *C.peninsularis* sp. nov.; and (3) Bintang Range and the surrounding lowlands, which includes the distribution of *A.larutensis* and NWL of *C.peninsularis* sp. nov.. Interestingly, Chan and Grismer (2021) also identified a Northwestern Region as a separate bioregion that includes the northwestern states of Penang, Kedah, and Perlis. *Cyrtodactyluspeninsularis* sp. nov. occurs in Kedah and Penang (Grismer 2011) but we were unable to include those populations in this study. Based on the congruence in the distribution patterns of *C.peninsularis* sp. nov. and bioregions identified by Chan and Grismer (2021), it is highly likely that the population genetic structure of *C.peninsularis* sp. nov. from the northwestern states of Kedah and Penang will reflect a similar regionalized separation. These data suggest that even though these sympatric species presumably shared the same or similar environmental history on the Thai-Malay Peninsula, differences in their phylogeographic structure are most likely due to their inherent biological differences, some being vagile forest dwellers, others being stream dwellers, and others being microhabitat specialists.

### ﻿Efficacy of analyses used herein

Davis et al. (2023) stated that “many species in the genus [*Cyrtodactylus*] are described by primarily relying on mitochondrial pairwise distance cut-offs that have not been rigorously tested…” First, a pairwise distance cut-off would not be used to “describe” a species but could be inappropriately used to delimit a species. Although their statement is likely to be true in a diagnostic sense, we know of no such case, nor were any citations provided. They also noted that “mitochondrial datasets can mislead delimitation decisions” and may only “represent population-level diversity”. It has been well-established that phylogenetic structure does not equal species level differences, and that statistically well-supported clades from any data set need evidence from the animals’ natural history (morphology, ecology, behavior, etc.), something not considered in Davis et al. (2024), to indicate these clades may be on separate evolutionary trajectories (Sukumaran and Knowles 2017). The mtDNA gene tree in Davis et al. (2023: Suppl. material 1) showed no discordance with their genomic tree and recovered the same major lineages (Fig. 2). However, these data are not corroborated with robust statistically defensible morphological analyses and lead the authors to naïvely posit that some morphological characters (they listed five) are also problematic because of interspecific variation. They went on to unwisely overstate that this variation would be the same for “many morphological characters” for “many other species complexes across the distribution of the genus”—which extends from South Asia to Melanesia (Grismer et al. 2021)—but provided no citations. The fact is that all morphological characters vary, but how this variation is treated is what matters. By not subjecting character variation in their datasets to basic univariate statistical analyses (e.g., student t-tests or ANOVAs) which can separate signal from noise, character variation may seem uninformative. Additionally, it matters which characters are used because one dataset does not fit all—especially within a genus as diverse (350+ species) as *Cyrtodactylus*. For example, many characters used to diagnose (*not* delimit) species among the karst-dwelling taxa of the *chauquangensis* group (Grismer et al. 2024) would be uninformative for diagnosing the terrestrial species in the *triedrus* group and vice versa (Amarasinghe et al. 2022; Agarwal et al. 2023). We emphasize here that by recognizing statistically significant morphological differences that correlate with strongly supported monophyletic geographically isolated mitochondrial (or nuclear) lineages, continues to move science forward by providing robustly corroborated testable hypotheses to be evaluated later with additional data sets and analyses (see Grismer et al. 2024a, 2025; Chhin et al. 2024; Neang et al 2024; and dozens of recent citations therein).

### ﻿Biogeography

As with many other species of lizards, the northern distribution of *Cyrtodactyluspeninsularis* sp. nov. terminates in the vicinity of the Kangar-Pattani Line (Grismer 2011; Quah and Anuar 2018; Quah et al. 2023 and references therein). Given that this is a major faunal, floristic, and climatic transition zone from aseasonal to seasonal evergreen tropical forest, tolerances of each species to different abiotic factors may affect the extent of their distribution. Because temperature and moisture levels affect the floral composition in this area (Baltzer et al. 2007), forest adapted species such as *C.peninsularis* sp. nov. and others may be more susceptible to changes in these variables across this transition zone than would rock-adapted species that usually do not depend on the associated forest for refuge or resources and rock habitats tend to harbor higher levels of humidity (e.g., Grismer 2011; Grismer et al. 2018).

## Supplementary Material

XML Treatment for Cyrtodactylus peninsularis

## ﻿Additional information

### Author ORCIDs

L. Lee Grismer https://orcid.org/0000-0001-8422-3698

Amanda Kaatz https://orcid.org/0009-0005-3444-4560

Jesse L. Grismer https://orcid.org/0000-0002-2542-5430

Eddie Nguyen https://orcid.org/0009-0003-0790-6817

Jeren J. Grergory https://orcid.org/0009-0003-3022-1621

Perry L. Wood Jr. https://orcid.org/0000-0003-3767-5274

Matthew L. Murdoch https://orcid.org/0000-0001-5914-6408

Shahrul Anuar https://orcid.org/0000-0003-0648-7318

Chan Kin Onn https://orcid.org/0000-0001-6270-0983

Muhamad A. Muin https://orcid.org/0000-0001-6712-265X

Parinya Pawangkhanant https://orcid.org/0000-0002-0947-5729

Chatmongkon Suwannapoom https://orcid.org/0000-0002-3342-1464

Nikolay A. Poyarkov https://orcid.org/0000-0002-7576-2283

Evan S. H. Quah https://orcid.org/0000-0002-5357-1953

### Data availability

All of the data that support the findings of this study are available in the main text or Supplementary Information.

## Supplementary materials

Supplementary material 1 Adjusted morphometric and mersitc charaters used in the MFA of the malayanus group Data type xlsx https://binary.pensoft.net/file/1351816This dataset is made available under the Open Database License (http://opendatacommons.org/licenses/odbl/1.0/). The Open Database License (ODbL) is a license agreement intended to allow users to freely share, modify, and use this Dataset while maintaining this same freedom for others, provided that the original source and author(s) are credited.L. Lee Grismer, Amanda Kaatz, Jesse L. Grismer, Eddie Nguyen, Jeren J. Grergory, Perry L. Wood Jr., Matthew L. Murdoch, Shahrul Anuar, Chan Kin Onn, Muhamad A. Muin, Parinya Pawangkhanant, Chatmongkon Suwannapoom, Nikolay A. Poyarkov, Evan S. H. Quah

Supplementary material 2 Adjusted morphometric and mersitc charaters used in the MFA *Cyrtodactylusconsobrinus*, *C.peninsularis* sp. nov. and *C.hutan* Data type xlsx https://binary.pensoft.net/file/1351817This dataset is made available under the Open Database License (http://opendatacommons.org/licenses/odbl/1.0/). The Open Database License (ODbL) is a license agreement intended to allow users to freely share, modify, and use this Dataset while maintaining this same freedom for others, provided that the original source and author(s) are credited.L. Lee Grismer, Amanda Kaatz, Jesse L. Grismer, Eddie Nguyen, Jeren J. Grergory, Perry L. Wood Jr., Matthew L. Murdoch, Shahrul Anuar, Chan Kin Onn, Muhamad A. Muin, Parinya Pawangkhanant, Chatmongkon Suwannapoom, Nikolay A. Poyarkov, Evan S. H. Quah

Supplementary material 3 Adjusted morphometric and mersitc charaters used for the lineages of *Cyrtodactyluspeninsularis* sp. nov. Data type xlsx https://binary.pensoft.net/file/1351818This dataset is made available under the Open Database License (http://opendatacommons.org/licenses/odbl/1.0/). The Open Database License (ODbL) is a license agreement intended to allow users to freely share, modify, and use this Dataset while maintaining this same freedom for others, provided that the original source and author(s) are credited.L. Lee Grismer, Amanda Kaatz, Jesse L. Grismer, Eddie Nguyen, Jeren J. Grergory, Perry L. Wood Jr., Matthew L. Murdoch, Shahrul Anuar, Chan Kin Onn, Muhamad A. Muin, Parinya Pawangkhanant, Chatmongkon Suwannapoom, Nikolay A. Poyarkov, Evan S. H. Quah

Supplementary material 4 Summary statistic of the adjusted morphometric and mersitic PCAs on the lineages of *Cyrtodactyluspeninsularis* sp. nov. Data type xlsx https://binary.pensoft.net/file/1351819This dataset is made available under the Open Database License (http://opendatacommons.org/licenses/odbl/1.0/). The Open Database License (ODbL) is a license agreement intended to allow users to freely share, modify, and use this Dataset while maintaining this same freedom for others, provided that the original source and author(s) are credited.L. Lee Grismer, Amanda Kaatz, Jesse L. Grismer, Eddie Nguyen, Jeren J. Grergory, Perry L. Wood Jr., Matthew L. Murdoch, Shahrul Anuar, Chan Kin Onn, Muhamad A. Muin, Parinya Pawangkhanant, Chatmongkon Suwannapoom, Nikolay A. Poyarkov, Evan S. H. Quah

Supplementary material 5 Additonal specimens examined Data type xlsx https://binary.pensoft.net/file/1351820This dataset is made available under the Open Database License (http://opendatacommons.org/licenses/odbl/1.0/). The Open Database License (ODbL) is a license agreement intended to allow users to freely share, modify, and use this Dataset while maintaining this same freedom for others, provided that the original source and author(s) are credited.L. Lee Grismer, Amanda Kaatz, Jesse L. Grismer, Eddie Nguyen, Jeren J. Grergory, Perry L. Wood Jr., Matthew L. Murdoch, Shahrul Anuar, Chan Kin Onn, Muhamad A. Muin, Parinya Pawangkhanant, Chatmongkon Suwannapoom, Nikolay A. Poyarkov, Evan S. H. Quah
